# Pancreaticoduodenectomy Combined with Vascular Resection and Reconstruction for Patients with Locally Advanced Pancreatic Cancer: A Multicenter, Retrospective Analysis

**DOI:** 10.1371/journal.pone.0070340

**Published:** 2013-08-02

**Authors:** Yi Gong, Leida Zhang, Tieying He, Jun Ding, Hongyu Zhang, Geng Chen, Dong Zhang, Zheng Wu, Qilong Chen, Haining Fan, Qi Wang, Ping Bie, Huaizhi Wang

**Affiliations:** 1 Institute of Hepatopancreatobiliary Surgery, Southwest Hospital, Third Military Medical University, Chongqing, P. R. China; 2 Department of Pancreatic Surgery, First Affiliated Hospital of Xinjiang Medical University, Urumqi, P. R. China; 3 Department of Hepatobiliary Surgery, The First Affiliated Hospital of Medical College, Xi’an Jiaotong University, Xi’an, Shaanxi Province, P. R. China; 4 Department of Hepatopancreatobiliary Surgery, Affiliated Hospital of Qinghai University, Xining, Qinghai, P. R. China; 5 The General Hospital of Ningxia Medical University, Yichuan, Ningxia, P. R. China; University of Nebraska Medical Center, United States of America

## Abstract

**Objective:**

The aim of this study was to present the therapeutic outcome of patients with locally advanced pancreatic cancer treated with pancreatoduodenectomy combined with vascular resection and reconstruction in addition to highlighting the mortality/morbidity and main prognostic factors associated with this treatment.

**Materials and Methods:**

We retrospectively analyzed the clinical and pathological data of a total of 566 pancreatic cancer patients who were treated with PD from five teaching hospitals during the period of December 2006–December 2011. This study included 119 (21.0%) patients treated with PD combined with vascular resection and reconstruction. We performed a detailed statistical analysis of various factors, including postoperative complications, operative mortality, survival rate, operative time, pathological type, and lymph node metastasis.

**Results:**

The median survival time of the 119 cases that received PD combined with vascular resection was 13.3 months, and the 1-, 2-, and 3-year survival rates were 30.3%, 14.1%, and 8.1%, respectively. The postoperative complication incidence was 23.5%, and the mortality rate was 6.7%. For the combined vascular resection group, complications occurred in 28 cases (23.5%). For the group without vascular resection, complications occurred in 37 cases (8.2%). There was significant difference between the two groups (p = 0.001). The degree of tumor differentiation and the occurrence of complications after surgery were independent prognostic factors that determined the patients’ long-term survival.

**Conclusions:**

Compared with PD without vascular resection, PD combined with vascular resection and reconstruction increased the incidence of postoperative complications. However, PD combined with vascular resection and reconstruction could achieve the complete removal of tumors without significantly increasing the mortality rate, and the median survival time was higher than that of patients who underwent palliative treatment. In addition, the two independent factors affecting the postoperative survival time were the degree of tumor differentiation and the presence or absence of postoperative complications.

## Background

Pancreatic cancer is a highly malignant tumor of the digestive system. In recent years, the incidence and mortality of pancreatic cancer have shown an upward trend worldwide [1], as the mortality rate of pancreatic cancer has leapt to fifth among cancer-related deaths [2]. Currently, the surgical removal of the entire tumor (en bloc resection of the pancreas and surrounding structures) is still considered the only possible clinical approach to cure pancreatic cancer [3]. However, because of its initial occult nature and biological behaviors, tumor invasions to the liver, lymph nodes, and the surrounding vessels and nerves, especially the superior mesenteric vein (SMV) and portal vein (PV), are commonly found [4]; meanwhile, the adjacent arteries, such as superior mesenteric artery (SMA) and common hepatic artery (CHA), are involved as well. It is relatively infrequent that the artery is involved while the vein is not infiltrated. On the other hand, segmental SMV/PV occlusion alone without SMA involvement is also rare [5]. Due to tumor invasions adjacent vascular, the surgical resection rate is only 15% to 20% [6]. Recently, the surgical approach of pancreatoduodenectomy (PD) combined with PV and SMV resection and reconstruction has been widely applied in clinical practice to remove the tumor completely. Therefore, vascular invasion is no longer a surgical contraindication, and the rate of surgical resection has greatly increased. Moreover, PD combined with vascular resection can account for 20% to 25% of the total cases of PD surgery in a number of the larger pancreas treatment centers [3], [7]–[8]. Several researchers have compared multiple aspects of PD combined with vascular reconstruction with PD surgery alone, including postoperative complications, mortality, survival, and surgery-related parameters. However, the existing data were mostly derived from single-center retrospective analyses, and the results obtained from different research centers are not exactly consistent with one another and sometimes even contradict one another [9], [10]. This study involved pancreatic cancer patients who were treated with PD combined with vascular resection in the pancreas treatment centers of multiple teaching hospitals in China. We performed statistical analyses focusing on demographics, operative factors, morbidity, mortality, and overall survival. We also investigated the primary factors that determined patient prognosis.

## Materials and Methods

### Ethics Statement

This retrospective study was approved by Institutional Review Board at Southwest Hospital, Third Military Medical University. All patients provided written informed consent.

### Patients

This study retrospectively collected the clinical and pathological data of a total of 566 pancreatic cancer patients who were treated in five teaching hospitals (the Southwest Hospital of the Third Military Medical University, Chongqing, China; the Xi’an Jiaotong University Affiliated Hospital, Shannxi, China; the Qinghai University School of Medicine, Qinghai, China; the Xinjiang Medical University, Xinjiang, China; and the Ningxia Medical College Affiliated Hospital, Ningxia, China) between December 2006 and December 2011. Among these patients, 119 were treated with PD combined with vascular resection and reconstruction, and the remaining patients underwent conventional PD. The follow-up time ranged from 9 to 79 months, the average follow-up time was 38.2 months, and the median follow-up period was 36.4 months. Overall, 25 patients were lost to follow-up, and the loss to follow-up rate was 4.4%. The preoperative examinations included disease history, physical examination, routine laboratory tests, examination of the tumor markers (carcino-embryonic antigen [CEA] and carbohydrate antigen [CA19-9]), chest radiograph, contrast-enhanced computed tomography (CT) or magnetic resonance imaging (MRI), and CT angiogram.

### Inclusion and Exclusion Criteria

When selecting cases, we mainly used the following principles: (1) before surgery, ECOG performance status of all patients is grade 0–2; (2) the preoperative imaging examination did not find distant metastases in the liver and abdominal cavity or ascites; (3)regarding to the vascular involved, patients that meet the M.D. Anderson criteria [5] were all suitable for PD with vascular resection; If the tumor encase the adjacent arteries (celiac axis, superior mesenteric artery, or both) or that occlude the SMV, PV, or SMPV confluence, it was considered contraindication of surgery and had be excluded; (4) the patients with systemic diseases, such as hypertension, diabetes and coronary heart disease, were included, but none of these patients had absolute surgical contraindications; (5) cases that had periampullary tumors in addition to pancreatic cancer, such as cancer of the lower bile duct, duodenal papilla cancer, and ampullary tumors, were not included in this study; (6) patients suffering from primary malignant tumors of other systems were also excluded from the study.

### Diagnosis of Vascular Invasion

We first determined a high suspicion of vascular invasion based on the preoperative imaging studies, such as enhanced CT or MRI and CT angiography. We use Loyer grade standard [11] recommended in *Pancreatic Cancer Diagnosis and Treatment Guidelines* of the Chinese Medical Association to grade the degree of vascular involvement. Preoperative CT of patients involved in the research suggested that the Loyer stages were all A-D. Second, we determined the possibility of vascular invasion intraoperatively based on the surgeon’s visual judgment. Finally, the presence and degree of vascular invasion were clarified by postoperative pathological diagnosis.

### Treatment Regimen

The treatment regimen mainly involved two stages. First, an en bloc excision of the tumor was performed. Because all cases included in the scope of this study had surgically resectable pancreatic cancer, en bloc resection of the pancreas and surrounding structures was used in all surgical regimens. All the patients underwent an open surgical resection rather than a laparoscopic resection. The specific surgical approach utilized PD with or without reserving the pylorus, and the criterion for determining whether the pylorus should be preserved depended primarily on the size of the cancer observed by the naked eye and the surgeon’s clinical experience. If the tumor and surrounding blood vessels (PV and SMV) were difficult to separate (e.g., because the tumor surrounded the blood vessels or because of inflammatory adhesion) during the surgery, vascular resection and reconstruction were performed to achieve complete removal of the tumor. To most surgeons, invasion of the artery is an absolute contraindication to surgical resection [12]–[13]. However, in our study, several such cases received PD with artery resection and reconstruction. If the preoperative imaging examinations did not reveal clear arterial invasion, the cancer was located close to any arteries, and the artery was partially wrapped by a tumor with a length less than 3 cm, PD combined with arterial resection and reconstruction was performed. However, to ensure safety, this procedure should only be performed in experienced hands.

The primary methods for vascular resection and reconstruction are as follows. 1. Partial wedge resection of the vascular wall, which is suitable for cases with a range of invaded blood vessels less than 1/3 of the circumference and with less severe invasion. After resection, suture and repair of the defective blood vessel wall with sutures or an artificial patch was used to repair the gap. 2. Venous end-to-end anastomosis, which is suitable for cases with a range of invaded blood vessels greater than 1/3 of the circumference and less than 5 cm. After resection of the invaded part, the two ends of the blood vessel were directly connected. 3. Artificial vascular graft. If the invaded blood vessel is longer than 5 cm, connecting the two ends directly after resecting the invaded part can be difficult; however, artificial blood vessels can be docked to the two ends and sutured. 4. Combined resection and reconstruction of multiple blood vessels. When the tumor has invaded a large area of the surrounding tissue, the invaded blood vessels must all be resected and reconstructed to completely remove the tumor. This approach includes the combined implementation of venous end-to-end anastomosis and end-to-side anastomosis of the splenic vein and PV and the combined implementation of an artificial vascular graft and the end-to-side anastomosis of the splenic artery and hepatic artery.

The scope of lymph node dissection included the dissection of the lymph and connective tissue between the inferior vena cava and the abdominal aorta, the dissection of the hilar soft tissue, and the resection of the soft tissue on the right side of the superior mesenteric artery together with duodenal mesentery [14].

Chemotherapy, the second stage of treatment, was started in the fifth week after the surgery. If the patients recovered smoothly and were in stable condition, they received gemcitabine systematic chemotherapy (1,000 mg/m^2^ on the 1st, 8th and 15th day; every 4 weeks for 1 course of treatment, with 6 courses of treatment for 1 cycle).

### Statistical Analysis

All data were analyzed using the Statistical Package for the Social Sciences (SPSS, version 18.0, SPSS Inc., Chicago, IL, USA). The measurement data were expressed as the mean, median and range, and the enumeration data were expressed as the count and percentage. Survival time was calculated from the day of surgery until the patient died or was lost to follow-up. The overall survival rate was calculated using the Kaplan-Meier estimator, and the differences among the different survival curves were compared using the log-rank test. The Cox regression model (Cox proportional hazards analysis) was used to determine the independent factors influencing overall survival. When the effects of the measurement data on overall survival were evaluated, all of the cases were grouped according to the quartile. The two independent samples t-test and chi-square test were used to compare the differences between PD combined with vascular resection and PD without vascular resection in aspects such as the operative time, blood loss, and basic patient information. All differences were examined using a two-sided test, and p<0.05 was considered to be statistically significant.

## Results

### Preoperative Information: Patient Profiles

A total of 566 patients were included in this study; of these, 119 (21.0%) received PD combined with vascular resection and reconstruction and 447 (79.0%) received traditional PD. The ages of the patients treated with PD combined with vascular resection were 30 to 82 years, with a mean age of 59 years. Detailed patient information is listed in Table S1. The main preoperative clinical manifestations included abdominal pain, jaundice, and weight loss. There were several other relatively rare clinical manifestations, including nausea, vomiting, diarrhea, elevated blood sugar, and itching skin. There was no significant difference in the preoperative patient profiles between the group treated with PD combined with vascular resection and the group without vascular resection. The detailed information of the patients’ tumor staging according to the pancreatic cancer TNM staging system standard (2010) of AJCC (American Joint Committee on Cancer) is shown in Table S1.

### Surgical Information: Surgical Method and Intraoperative Blood Loss

The 119 pancreatic cancer patients who received vascular resection and reconstruction included 13 patients who underwent pylorus-preserving PD; the remaining patients underwent PD surgery that did not preserve the pylorus. Regarding the vascular reconstruction methods, 18 cases (15.1%) underwent a partial resection and reconstruction of the blood vessel walls (lateral venorrhaphy), 51 cases (42.9%) underwent venous end-to-end anastomosis, 43 cases (36.1%) received artificial vascular grafts, and 7 cases (5.9%) underwent the resection and reconstruction of multiple blood vessels. Among the 447 cases treated with conventional PD, 100 underwent pylorus-preserving surgery; the remaining patients underwent PD surgery that did not preserve the pylorus. Among the 119 pancreatic cancer patients who received a combined vascular resection, 103 cases underwent a routine vein resection, 15 cases were treated with combined resection and reconstruction of the veins and arteries, and 1 case was treated with combined vascular resection and reconstruction and organ resection. Among the 15 pancreatic cancer patients who received combined artery resection and reconstruction, Four cases were treated with combined hepatic artery resection and reconstruction, 10 cases were treated with combined SMA resection and reconstruction, and 1 case was treated with combined splenic artery and common hepatic artery resection and reconstruction. One patient received a pancreaticoduodenectomy combined with right kidney resection because his right kidney suffered from the tumor invasion. The operative time ranged from 347 to 1210 min, with a median of 535 min. The detailed surgical information is listed in Table S2. The annual statistical results regarding the intraoperative blood loss of patients who underwent combined vascular reconstruction are listed in Table S3. According to the data listed in this table, the intraoperative blood loss decreased year after year, suggesting that the technique of combined vascular reconstruction has gradually matured. The median intraoperative blood loss in 2011 was 600 ml, and the intraoperative blood loss of PD without vascular reconstruction was 500 ml; there was no significant difference between the two values (p<0.001).

### Postoperative Information: Pathology Analysis, Survival, Mortality, and Incidence of Complications

#### Pathological analysis

The detailed pathology data are shown in Table S4. The maximum transverse diameter listed in the pathology report was used as a comparative indicator for tumor size. The tumor size of the group with combined vascular resection and reconstruction ranged from 1.6 cm to 15.0 cm, and the average size was 4.0±5.2 cm. The tumor size of the group without combined vascular resection and reconstruction ranged from 0.5 cm to 18 cm, and the average size was 3.0±1.7 cm. The p-value for the comparison between the two groups was less than 0.001, suggesting that the group with combined vascular reconstruction had significantly larger tumors. This finding is not difficult to understand because larger tumors are more likely to invade the surrounding blood vessels. As a result of aggressive and radical resection, the postoperative pathological reports revealed that the surgical margin in all the patients was negative. In terms of the degree of tumor differentiation, tumors with moderate differentiation were the majority, accounting for 70 (71.4%) and 259 (58.0%) of the cases in the two groups, respectively. The main histopathological type was ductal adenocarcinoma, accounting for 87.4% and 91.8%, respectively. There were 39 lymph node-positive cases (32.8%) among the 119 cases that received combined vascular resection and reconstruction, and there were 73 lymph node-positive cases (16.4%) in the group without vascular resection. A significant difference was found between the two (p = 0.003). The degree of malignancy in the patients in the vascular invasion group was higher than that of the patients in the nonvascular invasion group, and the patients in the vascular invasion group were also more prone to lymph node metastasis. The preoperative evaluation suggested that the patients in the vascular invasion group all had vascular invasion. However, in reality, imaging findings and intraoperative visual judgment are not the gold standards. The final pathological results suggested that 114 of the 119 cases that received vascular reconstruction did indeed have vascular invasion by the tumor, whereas the remaining 5 patients only had vascular inflammatory adhesions, not tumor invasion.

#### Postoperative complications

Postoperative complications were diagnosed in accordance with the standards of the International Study Group of Pancreatic Surgery [15], [16]. This study only considered a portion of the major complications; other complications, such as resection rupture and mild lung infection, were not included in the scope of this study. For the combined vascular resection group, complications occurred in 28 cases (23.5%). For the group without vascular resection, complications occurred in 37 cases (8.2%). There was a significant difference between the two groups (p = 0.001). Regarding the types of complications, intra-abdominal hemorrhage was the most common; the details are listed in Table S5.

#### Mortality

A total of eight patients (6.7%) in the combined vascular resection group died within 30 days after surgery, and 13 patients (3.0%) in the group without vascular resection died. There was no significant difference between the two groups (p = 0.236). Among the eight patients who died, three had a postoperative intra-abdominal hemorrhage, and the patients abandoned treatment for economic reasons; three died of postoperative cardiovascular accidents; and the remaining two patients died of postoperative multiple organ failure. Among the 13 patients in the group without vascular resection who died, 10 died of postoperative abdominal hemorrhage, and three died of acute liver and kidney failure. Overall, most of the patients who died had clear histories of hypertension or heart disease. Despite the preoperative assessment by specialists, the risk of cardiovascular accidents under stress is still high.

#### Sub-group analysis of arterial and venous resection

Among the 119 pancreatic cancer patients who received a combined vascular resection, 103 cases underwent a routine vein resection, 15 cases were treated with combined resection and reconstruction of the veins and arteries. One patient received a pancreaticoduodenectomy combined with right kidney resection. Among the 16 patients mentioned above, 6 patients (37.5%) had complications and 4 (25.0%) died after operation. In comparison, among the 103 patients, 22 patients (21.4%) had complications and 4 (3.9%) died after operation. Obviously, the complication rates of the two groups are not statistically significant (x^2^ = 2.0,*P*>0.05) while the mortality rates are remarkably different (x^2^ = 9.8,*P*<0.05).

#### Survival

**Figure 1:**
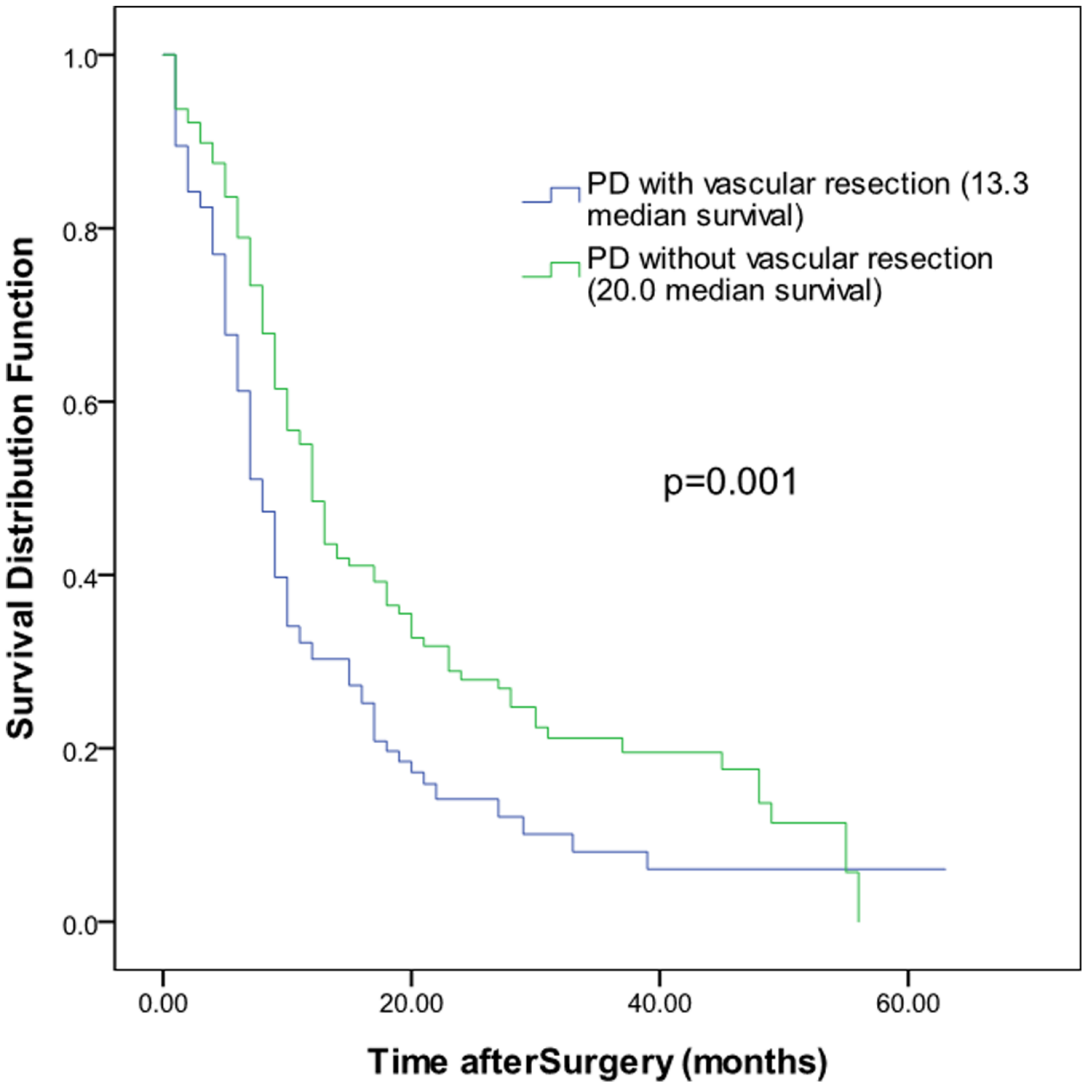
Comparison of the survival curves of the 119 cases treated with PD combined with vascular resection and reconstruction and those of the 447 cases treated with PD without vascular resection and reconstruction.

**Figure 2:**
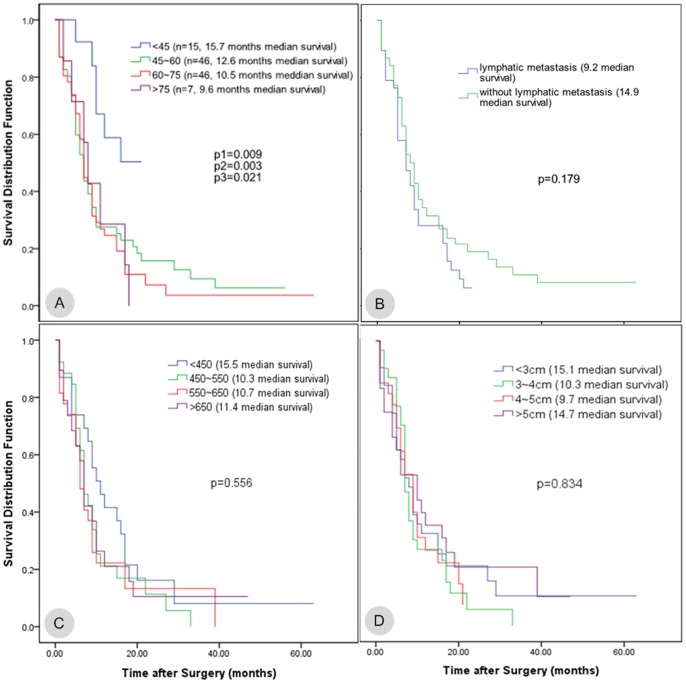
The relationship between the parameters of patients treated with combined vascular resection and reconstruction and the postoperative survival time. A. The patients were divided into four groups according to age: <45 years old, 45 to 60 years old, 60 to 75 years old, and >75 years old. The comparison between the first group and the rest of the groups yielded p<0.05. B. The patients were divided into two groups according to the presence or absence of lymph node metastasis. The median survival time of these two groups was 9.2 and 14.9 months, respectively (p = 0.179). C. The patients were divided into four groups according to the operative time: less than 450 min, 450 to 550 min, 550 to 650 min, and more than 650 min. The median survival time and the 95% confidence intervals were 15.5 (8.2, 22.8), 10.3 (6.8, 13.7), 10.7 (6.0, 15.4), and 11.4 (5.5, 17.3), respectively, with p = 0.556. D. The patients were divided into four groups according to tumor diameter: less than 3 cm, 3 to 4 cm, 4 to 5 cm, and greater than 5 cm. The median survival times and 95% confidence intervals were 15.1 (8.2, 22.0), 10.3 (7.4, 13.2), 9.7 (7.0, 12.5), and 14.7 (8.3, 21.2), respectively, with p = 0.834.

**Figure 3:**
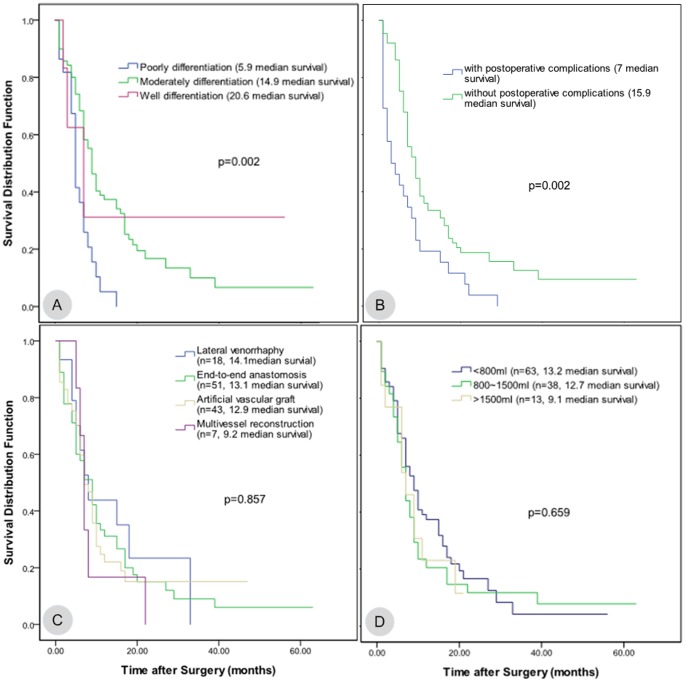
The relationship between the parameters of patients treated with combined vascular resection and reconstruction and postoperative survival time. A. The patients were divided into three groups based on the degree of differentiation. The median survival times and 95% confidence intervals for each group were 5.9 (4.3, 7.4), 14.9 (10.7, 19.2), and 20.6 (0, 45.1), respectively, with p = 0.002. B. The patients were divided into two groups with or without postoperative complications. The median survival times and 95% confidence intervals were 7 (4.0, 10.0) and 15.9 (11.6, 20.1), respectively, with p = 0.002. C. The patients were divided into four groups according to the different methods of vascular reconstruction. The median survival time and 95% confidence intervals for each group were 14.1 (7.1, 21.1), 13.1 (8.5, 17.8), 12.9 (8.0, 17.8), and 9.2 (4.0, 14.3), respectively, with p = 0.857. D. The patients were divided into three groups according to the volume of intraoperative blood loss: less than 800 ml, 800 to 1500 ml, and more than 1500 ml. The median survival times and 95% confidence intervals for each group were 13.2 (9.7, 16.8), 12.7 (7.1, 18.3), and 9.1 (5.4, 12.7), respectively, with p = 0.659.

The median overall survival time for the 119 patients with vascular reconstruction was 13.3 months, the 95% confidence interval was (10.1, 16.5), and the 1-, 2-, and 3-year survival rates were 30.3%, 14.1%, and 8.1%, respectively. For the 447 patients without combined vascular reconstruction, the average overall survival time was 20.0 months, the 95% confidence interval was (16.7, 23.3), and the 1-, 2- and 3-year survival rates were 55.1%, 27.9%, and 21.2%, respectively. The survival rates between the two groups were significantly different (log-rank, P<0.05) (Figure 1). The pancreatic cancer patients who received combined vascular resection and reconstruction were divided into four groups based on age: <45 years old, 45 to 60 years old, 60 to 75 years old, and >75 years old. The median survival time and the 95% confidence intervals for the four groups were 15.7 (12.5, 19.0), 12.6 (8.2, 17.0), 10.5 (6.7, 14.4), and 9.6 (5.0, 14.1), respectively. Log-rank testing found that the p values between the first group and the other three groups were 0.009, 0.003, and 0.021, respectively; all of these values were less than 0.05, indicating significant differences. The p values between the rest of the groups were all larger than 0.05 (Figure 2-A). Therefore, the survival time of patients younger than 45 years old was significantly longer than that of patients in the other age groups. The cases were divided into two groups based on the presence or absence of lymph node metastasis. The median survival time of the lymph node metastasis group was 9.2 months with a 95% confidence interval of (7.0, 11.3), and the median survival time of the group without lymph node metastasis was 14.9 months with a 95% confidence interval of (10.6, 19.3). There was no significant difference between the two groups (p = 0.179; Figure 2-B). In addition, the different vascular reconstruction approaches, intraoperative blood loss, operative time, tumor size, and other parameters had no significant effects on the patient survival rate (Figure 2-C, D; Figure 3-A, B, C, D). Using the Cox regression model to analyze the existing data, we found that the degree of tumor differentiation (relative risk, [RR] = 1.777, p = 0.028) and the presence or absence of postoperative complications (RR = 2.171, p = 0.003) were independent prognostic factors for the long-term survival of the pancreatic cancer patients who underwent combined vascular resection.

## Discussion

The morbidity and mortality associated with pancreatic cancer, a malignant tumor of the digestive system, have been increasing in recent years. In addition, the therapeutic outcome is still poor, which has attracted attention from an increasing number of researchers. The complete resection of the tumor by surgery is considered the only possible cure for pancreatic cancer. However, pancreatic tumors are adjacent to surrounding blood vessels and are prone to invading the PV and SMV. To achieve the complete removal of the tumor, PD combined with vascular resection and reconstruction must be performed. This surgical procedure is difficult and associated with a high-risk of mortality, but in recent years, the operative mortality has been reduced to approximately 5% [7], [17]–[18]. The mortality rate in the present study was basically consistent with the reported rate and did not differ significantly from that of PD without vascular resection and reconstruction. Additionally, most of the death cases had clear histories of heart disease. Therefore, for patients with a history of associated cardiovascular disease, more detailed and comprehensive preoperative assessments are necessary. However, the incidence of postoperative complications remains relatively high at approximately 30% to 50%, as indicated by previous reports [19]–[21]. In this study, the incidence of postoperative complications after PD combined with vascular resection and reconstruction was 23.5%, slightly lower than the values reported by other centers. The main type of complication was intra-abdominal hemorrhage. Compared with PD without vascular resection and reconstruction, PD with vascular resection and reconstruction had a higher incidence of postoperative complications, which showed a downward trend year by year as the surgical technology and expertise has matured. Although PD combined with vascular resection and reconstruction increased the incidence of postoperative complications in patients, this approach also achieved the complete removal of the tumor.

In this study, we also collected data on 48 pancreatic cancer patients who received a palliative operation (the combination of a cholangiojejunostomy and gastroenterostomy is what we called palliative operation) from January 2006 to June 2013. The reason these 48 patients received a palliative operation rather than a radical resection of tumor was not because they lost the chance to receive an en bloc resection, but mostly because they could not afford the expense of the operation or other reasons. The statistical analysis revealed that these patients’ average survival times and 95% confidence intervals were 9.6 (5.9, 13.4), significantly less than the median survival time of those patients who underwent a radical resection of tumor. The median survival time of the latter group was 13.3 months. Therefore, we believe that the application of vascular resection and reconstruction to achieve complete tumor resection has practical clinical significance.

Since Moore reported the world’s first case of PD combined with vascular resection and reconstruction in 1951 [22], the technique has significantly increased the resection rate in pancreatic cancer patients [23], [24]. However, as mentioned in the literature, PD combined with vascular resection and reconstruction does not improve patients’ long-term prognosis [18]. What are the factors affecting the prognosis of patients? This study mainly focused on and analyzed preoperative, intraoperative, and postoperative patient information, including patient age, tumor size, degree of differentiation, operative blood loss, operative time, lymph node status, method of vascular reconstruction, and postoperative complications. Positive lymph node metastasis and major perioperative complications have been reported to be the two main factors that affect patient survival time [25]. Other studies have reported that operative time and patient age are closely related to survival time [18]. In this study, we first used the log-rank test to individually select the parameters that significantly affected the patient survival time and identified the following three parameters: patient age, tumor differentiation degree, and the presence or absence of postoperative complications. We subsequently performed Cox regression analysis and concluded that only the degree of tumor differentiation and the presence/absence of postoperative complications were independent prognostic factors for the survival time of pancreatic cancer patients. Tumor size, the presence/absence of lymph node metastasis, operative time and intraoperative blood loss had no significant impact on the patients’ long-term survival. The risk of death among patients with serious postoperative complications was 2.2 times higher than that of patients without postoperative complications. Therefore, we have to minimize the incidence of postoperative complications as much as possible to prolong the patients’ postoperative survival time.

Additionally, we compared different vascular reconstruction methods. The median survival times for the partial resection of a blood vessel wall, venous resection combined with end-to-end anastomosis, artificial vascular graft, and resection and reconstruction of multiple blood vessels were 14.1 months, 13.1 months, 12.9 months, and 9.2 months, respectively. Although the differences in survival time were not significant, a trend can be observed that a milder degree of vascular invasion leads to longer patient survival times.

Among the 119 patients received pancreaticoduodenectomy combined with vascular resection, 103 patients received pancreaticoduodenectomy combined with venous resection (PV,SMV,spleen vein), 15 cases with arterial resection (SMA, CHA, splenic artery), and 1 with right kidney resection. After comparison, we found that there was no significant difference between the complication rates of PD combined with arterial and venous resection, while the mortality rates of the former is remarkably higher than the latter one. However, arterial invasion is not a contraindication of PD combined with vascular resection. Most physicians are cautious about the performance of PD combined with arterial resection and reconstruction, mainly because they suppose the patients with arterial invasion might have undergone tumor distant metastasis while venous invasion is a function of tumor size. But this viewpoint needs the support of fundamental research such as molecular biology and cytology. In addition, it should be pointed out that there are no large patient series on arterial resections in pancreatic surgery. Moreover, PD combined with arterial resection is difficult to operate, with great risk of hemorrhage. Therefore, it should be carried out safely in experienced hands.

CA19-9 is considered to be an important examination index affecting tumor recurrence and prognosis. Many scholars conducted a deep research on the relationship between CA19-9 and the occurrence and transfer of pancreatic cancer [26], and they indicated that if CA19-9 exceeds 150 U/ml, the tumor could likely not be resected [27]. In this study, we do not analyze CA19-9 in detail because the statistics and data available for it are not comprehensive enough. In addition, all of the patients included in this study received regular chemotherapy after the surgery (the chemotherapy regimens were performed as described above). In recent years, some studies have indicated that performing chemotherapy before the surgery on those pancreatic cancer patients who can undergo tumor resection can prolong their survival time. [28].

### Conclusions

Although PD combined with vascular resection and reconstruction increased the incidence of postoperative complications compared with PD without vascular resection, this approach achieved the complete removal of the tumor without significantly increasing the mortality rate. In addition, the incidence of postoperative complications showed a declining trend over the years as the surgical technology and expertise matured. Although PD combined with vascular resection and reconstruction completely resected the tumor, the median survival time of patients who underwent this procedure was still lower than that of pancreatic cancer patients who did not experience vascular invasion. However, the most important finding is that its median survival time is higher than the median survival time of patients who received palliative treatment. In other words, PD combined with vascular resection surgery has tangible clinical significance. By analyzing the relationship between various parameters of the patients and survival time, we clarified that the two independent factors affecting patient survival time were the degree of tumor differentiation and the presence/absence of postoperative complications. Reducing the incidence of postoperative complications can significantly increase patient survival time.

Because PD combined with vascular resection and reconstruction is a complicated procedure, its operative time and intraoperative blood loss are both greater than that of PD without vascular resection. This technique thus requires a surgeon with skilled surgical techniques and extensive clinical experience. In addition, follow-up studies should further improve the homogeneity of the subjects and should consider patient quality of life as a survey indicator to provide a theoretical basis for the clinical significance of PD combined with vascular resection and reconstruction.

## Supporting Information

Table S1 **Demographic characteristics of the patients and preoperative statistics.**
(DOCX)

Table S2 **Surgical information.**
(DOCX)

Table S3 **The annual statistical results for the intraoperative blood loss of patients who underwent combined vascular reconstruction** (median, ml).
(DOCX)

Table S4 **Comparison of the pathological data.**
(DOCX)

Table S5 **Incidences of postoperative complications and mortality.**
(DOCX)
