# Supplementary material for: Pancreaticoduodenectomy Combined with Vascular Resection and Reconstruction for Patients with Locally Advanced Pancreatic Cancer: A Multicenter, Retrospective Analysis
Source: PLoS One. 2013 Aug 2;8(8):e70340. doi: 10.1371/journal.pone.0070340 (PMC3732270; doi:10.1371/journal.pone.0070340)
Supplement: Table S1 — Demographic characteristics of the patients and preoperative statistics. (DOCX) [file pone.0070340.s001.docx]

**Table 1. Demographic characteristics of the patients and preoperative statistics**

|  | PD with vascular resection (n=119) | PD without vascular resection (n=447) | P Value |
| --- | --- | --- | --- |
| Age (yrs) |  |  |  |
| Range  Median | (30, 82)  59±11.5 | (37,81)  59±10.6 | p=0.873 |
| Sex |  |  |  |
| Male  Female | 72 (60.5%)  47 (39.5%) | 295(66.0%)  152(34.0%) | p=0.361 |
| Symptoms |  |  |  |
| Abdominal pain  Jaundice  Weight loss | 80 (82.5%)  50 (51.5%)  8 (8.2%) | 354(79.2%)  250(55.9%)  37(8.3%) | p=0.614  p=0.245  p=1.000 |
| CA19-9(U/ml) |  |  |  |
| Range  Median | (2,900)  172.0 | (2,1200)  106.0 | **-**  **-** |
| TNM Stage  ⅠStage  Ⅱ Stage  Ⅲ Stage  Ⅳ Stage |  |  | **-**  **-**  **-**  **-** |
|  | 4(3.4%)  78(65.5%)  35(29.4%)  2(1.7%) | 360(80.5%)  84(18.8%)  3(0.7%)  0 |  |
