# Supplementary material for: Pancreaticoduodenectomy Combined with Vascular Resection and Reconstruction for Patients with Locally Advanced Pancreatic Cancer: A Multicenter, Retrospective Analysis
Source: PLoS One. 2013 Aug 2;8(8):e70340. doi: 10.1371/journal.pone.0070340 (PMC3732270; doi:10.1371/journal.pone.0070340)
Supplement: Table S2 — Surgical information. (DOCX) [file pone.0070340.s002.docx]

**Table 2. Surgical information**

|  | PD with  vascular resection | PD without vascular resection | P value |
| --- | --- | --- | --- |
| Surgical options |  |  |  |
| Pylorus-preserving PD  Extended PD | 13 (10.9%)  106 (89.1%) | 100(22.4%)  347(77.6%) | p=0.122 |
| Vascular reconstruction technique |  |  |  |
| Lateral venorrhaphy  End-to-end anastomosis  Artificial vascular graft  Multivessel reconstruction | 18 (15.1%)  51(42.9%)  43 (36.1%)  7(5.9%) | -  -  -  - |  |
| Operative parameters |  |  |  |
| Intraoperative blood loss  (range and median, variance)  Operative time  (range and median, variance) | (100,7000) ml  800.0±1054.1 ml  (347, 1210) min  535.0±136.4 min | (150,2000) ml  500.0±348.8 ml  (258, 838) min  436±105.5 min | p=0.001  p=0.001 |
