# Supplementary material for: Pancreaticoduodenectomy Combined with Vascular Resection and Reconstruction for Patients with Locally Advanced Pancreatic Cancer: A Multicenter, Retrospective Analysis
Source: PLoS One. 2013 Aug 2;8(8):e70340. doi: 10.1371/journal.pone.0070340 (PMC3732270; doi:10.1371/journal.pone.0070340)
Supplement: Table S3 — The annual statistical results for the intraoperative blood loss of patients who underwent combined vascular reconstruction (median, ml). (DOCX) [file pone.0070340.s003.docx]

**Table 3. The annual statistical results for the intraoperative blood loss of patients who underwent combined vascular reconstruction (median, ml)***

| Year | 2006 | 2007 | 2008 | 2009 | 2010 | 2011 |
| --- | --- | --- | --- | --- | --- | --- |
| Blood loss (ml) | 1300 | 1000 | 1500 | 750 | 800 | 600 |
| Cases number | 3 | 13 | 21 | 21 | 28 | 28 |

*Note: There were 5 patients who received pancreaticoduodenectomy combined with vascular resection and reconstruction in 2012. This study didn’t completely count up the cases number of 2012, so the 5 patients’ blood loss amount is not given in Table 3.
