# Supplementary material for: Pancreaticoduodenectomy Combined with Vascular Resection and Reconstruction for Patients with Locally Advanced Pancreatic Cancer: A Multicenter, Retrospective Analysis
Source: PLoS One. 2013 Aug 2;8(8):e70340. doi: 10.1371/journal.pone.0070340 (PMC3732270; doi:10.1371/journal.pone.0070340)
Supplement: Table S4 — Comparison of the pathological data. (DOCX) [file pone.0070340.s004.docx]

**Table 4. Comparison of the pathological data**

|  | PD with  vascular resection | PD without  vascular resection | P value |
| --- | --- | --- | --- |
| Tumor size (cm) |  |  |  |
| Range  Median | (1.6, 15.0)  4.0±5.2 | (0.5, 18)  3.0±1.7 | P<0.001 |
| Histopathologic type |  |  |  |
| Well differentiated  Moderately differentiated  Poorly differentiated | 6 (6.1%)  70 (71.4%)  22 (22.4%) | 104 (23.2%)  259 (58.0%)  84(18.8%) |  |
| Histopathological types |  |  |  |
| Ductal adenocarcinoma  Mucinous adenocarcinoma  [Squamous](app:ds:squamous) or [adenosquamous](app:ds:adenosquamous) [carcinoma](app:ds:carcinoma)  Acinar cell carcinoma  Sarcomatoid carcinoma  [Neuroendocrine](app:ds:neuroendocrine) [carcinoma](app:ds:carcinoma) | 104 (87.4%)  5 (4.2%)  3 (2.5%)  4 (3.4%)  1 (0.8%)  2 (1.7%) | 410 (91.8%)  17 (3.7%)  7 (1.5%)  7(1.5%)  0  6 (1.3%) |  |
| Metastasis |  |  |  |
| Lymphatic metastasis (+, percentage)  Nerve invasion (+, percentage)  Vascular invasion(+,percentage) | 39, 32.8%  9, 7.6%  114, 95.8% | 73, 16.4%  13, 3.0%  0 | p=0.003  p=0.152 |
