# Supplementary material for: Pancreaticoduodenectomy Combined with Vascular Resection and Reconstruction for Patients with Locally Advanced Pancreatic Cancer: A Multicenter, Retrospective Analysis
Source: PLoS One. 2013 Aug 2;8(8):e70340. doi: 10.1371/journal.pone.0070340 (PMC3732270; doi:10.1371/journal.pone.0070340)
Supplement: Table S5 — Incidences of postoperative complications and mortality. (DOCX) [file pone.0070340.s005.docx]

**Table 5. Incidences of postoperative complications and mortality***

|  | PD with  vascular resection | PD without  vascular resection | P value |
| --- | --- | --- | --- |
| Overall complications (n, %)  Intra-abdominal hemorrhage (n, %)  Pancreatic fistula (n, %)  Biliary leak (n, %)  Delayed gastric emptying (n, %)  Thrombosis (n, %)  Stress ulcer (n, %)  Wound infection (n, %)  Intra-abdominal infection (n, %)  Hepatic/renal failure  Reoperation (n, %)  Surgical mortality (n, %) | 28, 23.5%  14, 50%  7, 25%  5, 17.9%  0  1, 3.6%  3, 10.7%  2, 7.1%  8, 28.6%  3, 10.7%  9, 32.1%  8, 6.7% | 37, 8.2%  13, 36.4%  3, 9.1%  0  3, 9.1%  0  13, 36.4%  7, 18.2%  7, 18.2%  7, 18.2%  10, 27.3%  13, 3.0% | p=0.001  p=0.236 |

*Note：The diagnosis of pancreatic fistula, delayed gastric emptying, and intra-abdominal hemorrhage was based on the criteria developed by the International Pancreatic Surgery Study Group [8, 9]. Postoperative mortality referred to patients who died within 30 days (not including 30 days) after surgery.
